# Mean amplitude of low frequency fluctuations measured by fMRI at 11.7 T in the aging brain of mouse lemur primate

**DOI:** 10.1038/s41598-023-33482-8

**Published:** 2023-05-17

**Authors:** Clément M. Garin, Marc Dhenain

**Affiliations:** 1 UMR 9199, Neurodegenerative Diseases Laboratory, Centre National de la Recherche Scientifique (CNRS), Université Paris-Sud, Université Paris-Saclay, 18 Route du Panorama, 92265 Fontenay-aux-Roses, France; 2 Commissariat à l’Energie Atomique et aux Energies Alternatives (CEA), Direction de la Recherche Fondamentale (DRF), Institut François Jacob, MIRCen, 18 Route du Panorama, 92265 Fontenay-aux-Roses Cedex, France

**Keywords:** Neural ageing, Neural circuits

## Abstract

Non-human primates are a critical species for the identification of key biological mechanisms in normal and pathological aging. One of these primates, the mouse lemur, has been widely studied as a model of cerebral aging or Alzheimer's disease. The amplitude of low-frequency fluctuations of blood oxygenation level-dependent (BOLD) can be measured with functional MRI. Within specific frequency bands (e.g. the 0.01–0.1 Hz), these amplitudes were proposed to indirectly reflect neuronal activity as well as glucose metabolism. Here, we first created whole brain maps of the mean amplitude of low frequency fluctuations (mALFF) in young mouse lemurs (mean ± SD: 2.1 ± 0.8 years). Then, we extracted mALFF in old lemurs (mean ± SD: 8.8 ± 1.1 years) to identify age-related changes. A high level of mALFF was detected in the temporal cortex (Brodmann area 20), somatosensory areas (Brodmann area 5), insula (Brodmann areas 13–6) and the parietal cortex (Brodmann area 7) of healthy young mouse lemurs. Aging was associated with alterations of mALFF in somatosensory areas (Brodmann area 5) and the parietal cortex (Brodmann area 7).

## Introduction

Human life expectancy has dramatically increased during the last century. This comes with an increased risk for cerebral alterations leading to neurodegenerative diseases or mild cognitive/motor impairments that impair daily living. Sensorimotor function impairment is one of the stereotypical characteristics of mammalian cerebral aging. It includes slower performance of visual and motor tasks^1^ such as walk speed^2^, movement coordination or dexterity^3^. In addition, higher functions such as thinking, memorizing, and speech capacity decline with aging^4^. These functional deficits occur alongside considerable brain alterations that include fiber demyelination^5^ and cortical thinning^6^. In non-human primates such as chimpanzees, white matter deterioration was observed, confirming prior results obtained in humans^7^. In addition, several investigations in mammalian models have shown an impact of aging on synapses and dendrites^8,9^. Monitoring functional impairments associated with cerebral ageing with translational imaging methods is critical to understand mechanisms leading to brain alterations and develop new treatments.

Measures of cerebral glucose metabolism using the radiolabeled glucose analog ^18^F-fluorodeoxyglucose (FDG) detected by Positron Emission Tomography (PET) imaging is a largely used marker to investigate brain function in elderlies^10,11^ and in the mouse model of Alzheimer's disease^12^. The use of PET is however restricted as this requires using radioactive compounds and is limited for animal studies by its low resolution when compared to functional magnetic resonance imaging (fMRI). In consequence, magnetic resonance imaging (MRI) is an interesting alternative to PET.

Low-frequency oscillations (LFO) of blood-oxygen level dependent (BOLD) signal reflects the total power of BOLD signal within the frequency range between 0.01 and 0.1 Hz. The amplitude of low-frequency fluctuations (ALFF) is expected to reflect neuronal activity^13^ and was associated with markers of glucose metabolism^14^. Thus, it has been proposed as an MRI-based method to evaluate brain function^13,15^ and could be a promising radioactive-free alternative to FDG-PET. Studies in humans have shown that ALFF is negatively correlated with age in several brain regions such as the supplementary motor area, pre-supplementary motor area (Brodmann area (BA) 6), anterior cingulate cortex (BAs 24, 32, 33), bilateral dorsal lateral prefrontal cortex (BAs 9, 46), right inferior parietal lobule (BAs 39, 40), and posterior cingulate cortex (BAs 23, 31)^13,15^. However, healthy aging effects on ALFF indexes remain to our knowledge, unexplored in non-human mammalians.

The mouse lemur (*Microcebus murinus*) is a primate attracting increased attention in neuroscience research and particularly for aging studies. This small animal (typical length 12 cm, 60–120 g weight) has a decade-long lifespan^16^ and is a model for studying cerebral ageing^17^ and Alzheimer's disease^18^. This animal was used to establish the impact of prediabetes in the brain^19^ as well as to evaluate interventions modulating cerebral ageing process^16^. The aim of the current study was thus to characterize ALFF in this primate in normal and ageing conditions. We described regional differences of ALFF signal and showed aged-related changes in specific brain regions.

## Materials and methods

### Animals and breeding

The guidelines of the European Communities Council directive (2010/63/EU) were followed when conducting this study. Our protocol was authorized by the local ethics committees CEtEA-CEA DSV IdF (authorizations 201506051736524 VI (APAFIS#778)). We originally included 33 mouse lemurs (21 males and 12 females) in our study (Table 1). They were bred in our laboratory (Molecular Imaging Research Center, CEA, Fontenay-aux-Roses) after being born at the CNRS/Brunoy, MNHN’s France, laboratory breeding colony (UMR 7179 CNRS/MNHN). Four animals that displayed MR images with artefact or brain lesions were removed from the study.

**Table 1:** Mouse lemur cohort and physiological parameters.

Subject	Sex	Age (years)	Animal rejected	Temperature (°C)	Isoflurane level (%)
1	MALE	1.3	NA	32	1.25
2	MALE	1.3	NA	32.2	1.25
3	MALE	1.4	NA	32	1.25
4	MALE	1.4	NA	32	1.25
5	MALE	1.4	NA	31.9	1.25
6	MALE	1.4	NA	32	1.25
7	MALE	1.5	NA	32	1.25
8	MALE	2.3	NA	32	1.25
9	MALE	2.4	NA	32	1.25
10	MALE	2.4	NA	32	1.4
11	MALE	2.4	NA	31.9	1.35
12	MALE	2.5	NA	32	1.25
13	FEMALE	2.9	NA	32	1.15
14	FEMALE	3.1	NA	31.9	1.25
15	FEMALE	3.2	Cerebral lesion	32	1.25
16	FEMALE	3.8	Cerebral lesion	32.5	1.2
17	MALE	8.0	NA	32	1.4
18	FEMALE	8.0	NA	32	1.5
19	MALE	8.0	NA	32	1.25
20	MALE	8.0	NA	32	1.25
21	FEMALE	8.0	NA	32	1.5
22	MALE	8.0	NA	32	1.5
23	FEMALE	8.0	NA	32	1.5
24	MALE	8.1	NA	32	1.5
25	FEMALE	8.1	NA	32	1.25
26	MALE	8.7	NA	32	1.25
27	MALE	8.7	NA	32	1.25
28	MALE	8.8	Artefact	32	1.5
29	FEMALE	8.8	NA	32	1.25
30	MALE	10.3	NA	32	1.25
31	FEMALE	10.4	NA	32	1.25
32	FEMALE	10.8	Artefact	32	1.25
33	FEMALE	10.8	NA	32.1	1.25

*The "young lemur cohort"* consisted of 14 animals with an age range of 1.3 to 3.8 years old (mean ± SD: 2.1 ± 0.8 years).

*The "old lemur cohort"* consisted of 15 animals with an age range of 8.0 to 10.8 years old (mean ± SD: 8.8 ± 1.1 years).

The animals were housed in cages with one or two lemurs, enrichment for jumping and hiding, temperatures between 24 and 26 degrees Celsius, a relative humidity of 55 percent, and seasonal illumination (summer: 14 h of light, 10 h of darkness; winter: 10 h of light, 14 h of darkness). Food consisted of fresh apples and a handmade blend of bananas, cereals, eggs, and milk. Water supply for animals was freely accessible. None of the animals had ever taken part in invasive research or pharmaceutical trials before.

### Animal preparation and MRI acquisition

To ensure animal stability during the experiment, all animals were scanned once while under isoflurane anesthesia at 1.25–1.5% in air, with respiratory rate monitoring. A 32 °C air heating system was used to maintain body temperature, causing mouse lemurs to go into a state of natural torpor^20^. The body temperature was maintained using a heating pad and measured using a rectal thermometer. The benefit of this is that it prevents reawakening while maintaining a low anesthetic level. Animals were scanned on an 11.7 Tesla Bruker BioSpec MRI machine (Bruker, Ettlingen, Germany) running ParaVision 6.0.1 with a volume coil for radiofrequency transmission and a quadrature surface coil for reception (Bruker, Ettlingen, Germany). We acquired anatomical images with a T2-weighted multi-slice multi-echo (MSME) sequence: TR = 5000 ms, TE = 17.5 ms, FOV = 32 × 32 mm, 75 slices of 0.2 mm thickness, 6 echoes, 5 ms IET, resolution = 200 µm isotropic, acquisition duration 10 min. We acquired resting state time series with a gradient-echo echo planar imaging (EPI) sequence: TR = 1000 ms, TE = 10.0 ms, flip angle = 90°, repetitions = 450, FOV = 30 × 20 mm, 23 slices of 0.9 mm thickness and 0.1 mm gap, resolution = 312.5 × 208.33 × 1000 µm, acquisition duration 7m30s. During the acquisition, the animals were head-fixed using ear bars to minimize head motion. The total duration of anesthesia was approximately one hour.

### MRI pre-processing

Data from scanners was exported as DICOM files and then changed to NIfTI-1 format. Then, using the Python program sammba-mri (SmAll MaMmals Brain MRI), spatial preprocessing was carried out^21^, http://sammba-mri.github.io) and we used nipype for pipelining^22^, leverages AFNI^23^ for most steps and RATS^24^ for “skullstripping”. A study template was made using the mutual registration of anatomical MR images. Images were then registered to a high-resolution anatomical mouse lemur template, built for our previously published functional atlas^25^. Motion, B0 distortion, and slice timing (interleaved) were removed from resting state MR images (per-slice registration to respective anatomical images). Using sequential applications of the transformations from individual anatomical images to the study template and then the transformations from study template to the mouse lemur atlas, all the MR images were placed into the same space. Functional images were further pre-treated using AFNI afni_proc.py^23^. fMRI images were smoothed (0.9 mm), bandpass filtered, detrend corrected (0.01–0.1 Hz) as well as slice timing and motion corrected. TRs with excessive motion of 0.07 mm or where too many voxels were flagged as outliers by 3dToutcount (AFNI), were censored. To ensure steady-state magnetization, the first five volumes were not included in the study. Note that standardization of fMRI pre-processing remains ongoing in human fMRI as in 2012, Carp et al. found 207 different analysis pipelines in 241 studies, suggesting that almost every publication uses a unique analysis pipeline^26^. However, significant efforts have been made through standardized protocol as proposed by fMRIPrep^27^. In rodents, a significant portion (26%, without taking into account pre-processing parameters) of rodent experiments used a specially designed pre-processing pipeline^28^. This suggests that standardization in non-human fMRI is still a far-off goal likely due to the adaptation of specific pre-processing parameters to each species’ physiology.

### mALFF calculation and extraction

LFO measures were performed using the fast Fourier transform index: amplitude of low-frequency fluctuation (ALFF)^29^. As ALFF is sensitive to the scale of raw signal and the unit of BOLD signal is arbitrary, the original ALFF value is not adapted for comparisons between animals. In addition, ALFF can be susceptible to signal fluctuations caused by physiological noise unrelated to brain activity^13^. A standardization procedure has been proposed by dividing the signal of each voxel by the global mean ALFF in each animal^30^. The newly calculated index is called mean ALFF (mALFF). mALFF indexes were calculated for each voxel of the pre-processed EPI images in the low-frequencies range 0.01 to 0.1 Hz using the function “3dLombScargle” and “3dAmpToRSFC” from AFNI^23^. The mALFF signal of each voxel was extracted within the different regions based on the anatomical atlas^31^ using NiftiLabelsMasker from Nilearn^32^.

### Statistical analysis

Voxel wise analysis was performed using 3dttest++ from AFNI^23^ and a clustering approach (-Clusterize) for multiple comparisons. This method is routinely used as statistical correction technique for multiple comparisons in fMRI research^33^. This method is based on the fact that fMRI voxels are not entirely independent and propose clusters of voxels to be evaluated for significance rather than each one separately. The outcome of this analysis (3dttest++) associates a cluster size to an uncorrected significant threshold value in a table form. In our study, we took the highest p-value (0.05) that was associated with a cluster size of 856 voxels. Analysis space was reduced by thresholding the average mALFF map to the 20% highest voxels. Then, using “map_threshold” from nilearn^32^, we extracted from the z-map, any cluster of statistical voxels superior to 856 associated to p < 0.05.

## Results

### mALFF in young mouse lemurs

**Figure 1:**
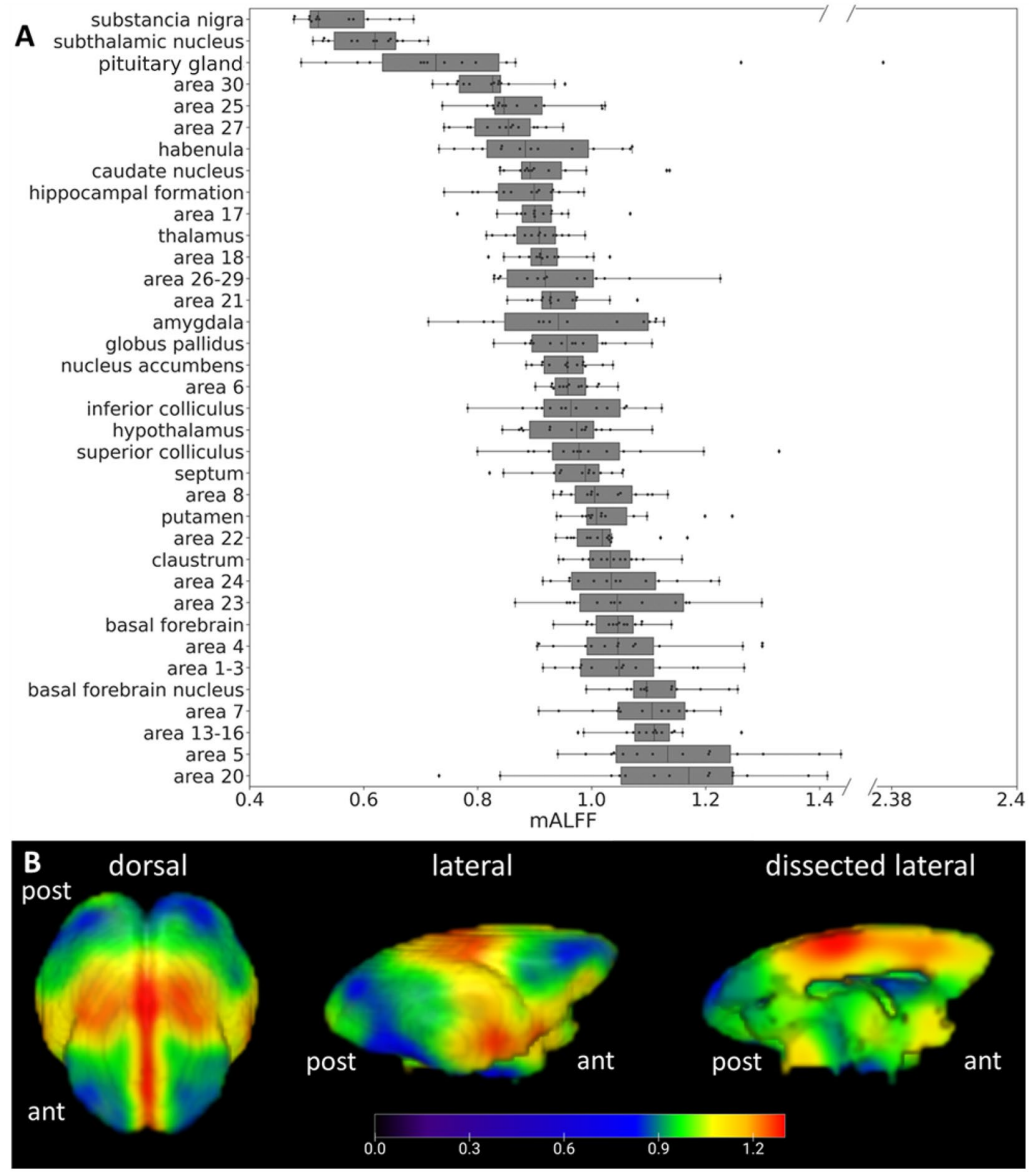
Distribution of mALFF in the brain of healthy young mouse lemurs. (**A**) mALFF in different brain regions of healthy young adult mouse lemurs (n = 14) ranked based on their group median value. Regional average of mALFF was extracted using a reference anatomical atlas of the mouse lemur after co-registration to the anatomical reference template^31^. Elevated mALFF is observed within regions encompassing cortical BAs 20, 5, 13–16, 7, 1–3, 4, 23, 24 and the basal forebrain. (**B**) Healthy distribution of mALFF signal variations displayed as a 3D brain surface average-map. ant: anterior part of the brain, post: posterior part of the brain.

mALFF maps were recorded from 14 young mouse lemurs. Individual mALFF maps of each animal were averaged to produce 3D maps of the group (Fig. 1). Automatic extractions of the mALFF signal were then performed in various cerebral regions by using a reference anatomical atlas (^31^; Fig. 1A). The highest cortical signal was observed in the temporal cortex (Brodmann area (BA) 20 *i.e.* secondary visual area, expected to be involved in visual processing and recognition memory), parietal cortex (parietal regions as BA 5 *i.e.* covering the superior parietal lobule (and a portion of the postcentral gyrus) involved in the representation of spatial information of limb movement^34^. High signal was also detected in the primary somatosensory cortex (BA 1–3) and the primary motor cortex (BA 4). More integrative areas such as the insula (BA 13–16) and in the cingulate cortex (BA 23 and 24) also displayed high signal. In subcortical areas, the basal forebrain exhibited the most elevated signal when compared to the rest of the brain. Conversely, cortical regions such as BA 30 (agranular retrolimbic area involved in vision) and the adjacent BA 27 (area presubicularis involved in vision) or BA 25 (antero-ventral part of the cingulate cortex) displayed the lowest levels of mALFF. Subcortical regions such as substantia nigra, subthalamic nucleus or pituitary gland also displayed the lowest levels of mALFF.

### Age-related changes of mALFF

**Figure 2:**
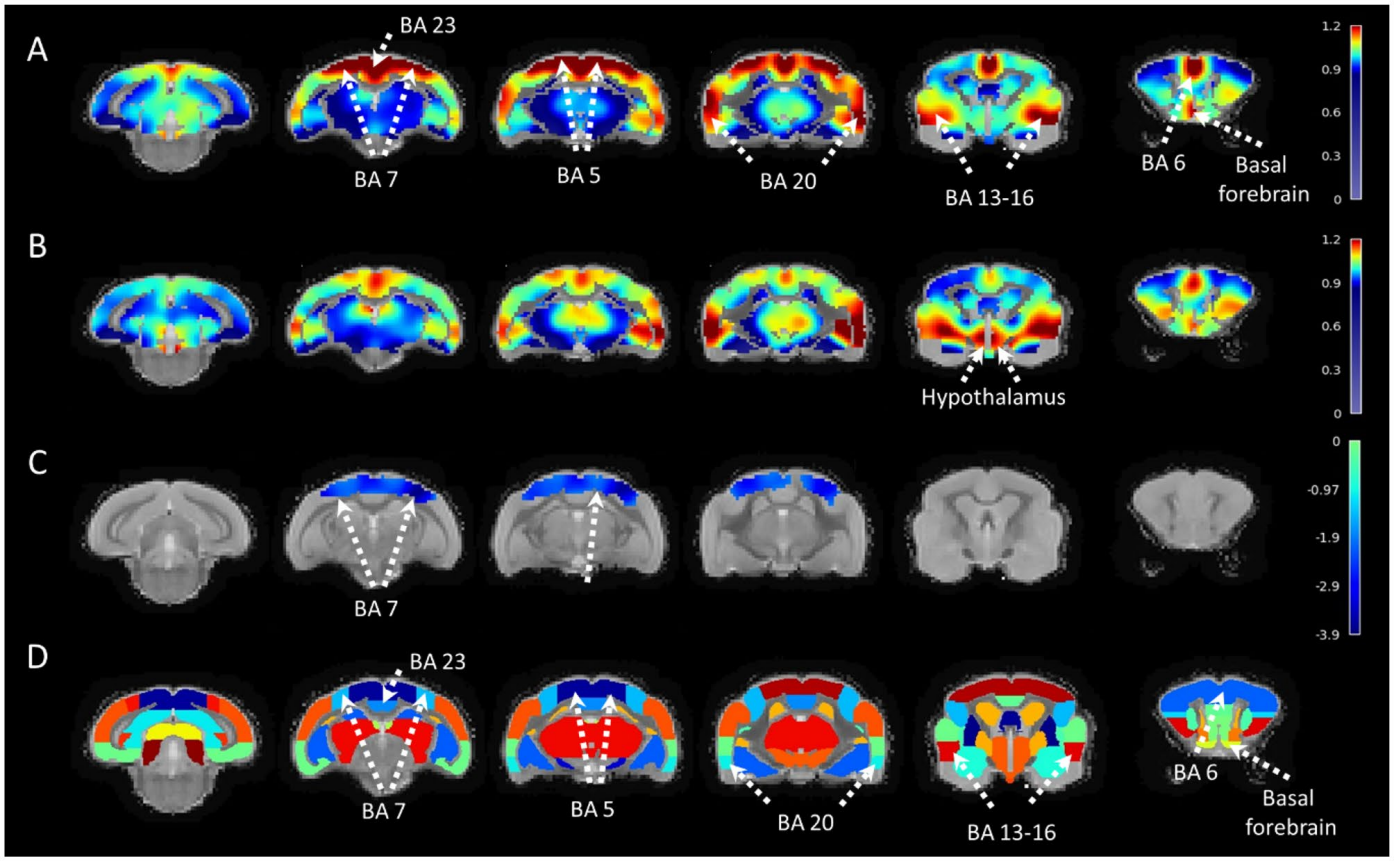
Statistical comparisons of the mALFF contrast in young and old mouse lemurs. Average mALFF maps in young (**A**) and old mouse lemurs (**B**) displayed as 2D coronal slices of the brain from posterior (left) to anterior (right) locations. Maps are shown as a colour-coded overlay on the mouse lemur reference template. Differences of mALFF between young and old mouse lemurs were assessed using a t-test (AFNI;^23^) associated to a clustering approach. A significant statistical decline of the mALFF signal was detected using voxelwise analysis in the BAs 5 and 7 (**C**). Anatomical regions of the mouse lemur atlas corresponding to the displayed sections (**A**–**C**) are shown in (**D**)^31^. BA: Brodmann area. Scale bars in **A**–**C**: mALFF contrast, in (**D**): z-score.

mALFF measures were performed in 15 mouse lemurs (old cohort) and compared to the previously studied young lemurs. Average mALFF maps are displayed in Fig. 2A for young mouse lemurs and in Fig. 2B for old mouse lemurs. Comparison of average mALFF maps between young and old lemurs indicates lower mALFF in the parietal cortex of aged animals and a trend towards higher signal in the hypothalamic regions. Voxelwise analysis between groups revealed a significant loss of BOLD signal amplitude in parietal regions involving (BA 5 and BA 7) of old animals (Fig. 2C, D). We used the same analysis to evaluate differences between males and females. No significant cluster was detected. In the absence of a significant effect of gender on mALFF index, the variable “gender” was not considered as a cofactor.

## Discussion

### Cerebral distribution of mALFF index in mouse lemurs

This study evaluated mALFF index in mouse lemur primates at a high field (11.7 T). The evaluation of mALFF in a young cohort provided 3D maps of the normal distribution of the mALFF indexes. Highest levels of mALFF were detected in cortical structures involved in high-order visual processing (BA 20 in temporal region), somatosensory (BA 5 in parietal region), and in integrative regions involved in visuo-motor coordination (BA 7 in parietal region). High signal was also detected in the primary somatosensory cortex (BA 1–3) and the primary motor cortex (BA 4). More integrative areas such as the insula (BA 13–16) and the cingulate cortex (BA 23 and 24) also displayed a high signal.

### Age-related changes of mALFF index

As a second part of the study, we assessed age-related changes of mALFF. In humans, healthy ageing mainly affects ALFF in brain regions involved in motor function such as the supplementary motor area or the pre-supplementary motor area. More integrative regions such as the anterior cingulate cortex, the dorso-lateral prefrontal cortex, the posterior cingulate cortex, and the inferior parietal lobule are also impaired^35^. In mouse lemurs, the only regions significantly affected by healthy ageing were those involved in visuo-somatosensory-motor function (parietal regions as BA 5 i.e. covering the superior parietal lobule (and a portion of the postcentral gyrus) involved in the representation of spatial information of limb movement^34^ and BA 7 i.e. an integrative area involved in visuo-motor coordination). These two regions are involved in somatosensory processing, in movement such as grasping^36^ or object location^37^. These alterations could participate in the described visuo-somatosensory-motor alterations reported in aged lemurs^38^.

Atrophy detected in mouse lemurs impacts the whole brain in the latest stages of ageing but the most prominent atrophied areas include the insular (BAs 13–16), frontal (BA 6), parietal (BAs 5, 7), occipital (BAs 17, 18), inferior temporal (BAs 21, 28) and cingulate cortices (BAs 23, 24, 25)^17,31^. In consequence, an ageing effect can be detected with mALFF and anatomical measures in both BAs 5 and 7 but BAs such as 17, 18, 13–16 are impacted only by atrophy and not by mALFF changes. A better understanding of the significance of mALFF is now needed to further interpret mALFF alterations during aging.

In previous studies, ALFF or fractional ALFF were used to evaluate the effect of simian immunodeficiency virus^39^, spinal injury^40^ or anesthesia^41^ in macaques. In mice, Huntington‐related pathological effects were detected using ALFF^42^. In rats, various effects of stress could also be evaluated using ALFF^43,44^. By showing age-related changes, the current study increases the range of ALFF changes detected in mammals in pathological situations.

Our results cannot be easily be interpreted within the context of the current literature. Indeed, to our knowledge no study evaluated the impact of aging on mALFF in other non-human primate species. In addition, our results do partially fit with the results obtained in humans^35^ showing mostly an effect of aging on fractional ALFF in prefrontal regions and in the posterior cingulate cortex (areas particularly involved in the default mode network).

### Limitations

Anesthesia is known to interact with brain function. For example, Isoflurane changes functional connections between brain regions in marmosets^45^. In addition, Wu et al. showed that across functionally related but different S1 subregions, isoflurane elicits comparable dose-dependent suppressive effects on the power of rsfMRI signals and local fine-scale functional connectivity^41^. Thus, one of the possible limitations of the study is anesthesia, as it could impact the mALFF signal and change the distribution of mALFF in the brain of healthy young mouse lemurs. Future studies will thus have to be conducted to investigate the impact of anesthesia on mALFF.

Comparisons between young and old animals revealed local age-related differences of mALFF. As both groups were anesthetized, this difference is expected to reflect the ageing effect. We can however not exclude that the suppressive effect of anesthesia may have decreased our ability to detect mALFF changes in some regions affected by ageing.

## Conclusion

In conclusion, this study provides evidences suggesting that mALFF can be measured in the whole brain of mouse lemurs and can detect aged-related changes. It highlights mALFF as a tool for the exploration of the cerebral function in mammals as well as an interesting candidate for the longitudinal follow up of age-related cerebral dysfunction in animal models.

## Data Availability

Raw MRI data in mouse lemurs are available upon request following a formal data sharing agreement required by authors’ institution. The template and atlas used in this study maps are available for download in NIfTI-1 format at https://www.nitrc.org/projects/mouselemuratlas.

## Code availability

All the software used to perform the analysis are publicly available. In particular, sammba-mri, the code developed by our team for spatial pre-processing is available at http://sammba-mri.github.io. All the third party codes used in the article are cited.

## References

[1] de Bruin N, Bryant DC, MacLean JN, Gonzalez CL (2016). Assessing visuospatial abilities in healthy aging: A novel visuomotor task. Front. Aging Neurosci..

[2] Bohannon RW (1997). Comfortable and maximum walking speed of adults aged 20–79 years: Reference values and determinants. Age Ageing.

[3] Serrien DJ, Swinnen SP, Stelmach GE (2000). Age-related deterioration of coordinated interlimb behavior. J. Gerontol. B Psychol. Sci. Soc. Sci..

[4] Blazer DG, Yaffe K, Karlawish J (2015). Cognitive aging: A report from the Institute of Medicine. JAMA.

[5] Marner L, Nyengaard JR, Tang Y, Pakkenberg B (2003). Marked loss of myelinated nerve fibers in the human brain with age. J. Comp. Neurol..

[6] Salat DH (2004). Thinning of the cerebral cortex in aging. Cereb. Cortex.

[7] Chen X (2013). Brain aging in humans, chimpanzees (Pan troglodytes), and rhesus macaques (*Macaca mulatta*): Magnetic resonance imaging studies of macro- and microstructural changes. Neurobiol. Aging.

[8] Dickstein DL, Weaver CM, Luebke JI, Hof PR (2013). Dendritic spine changes associated with normal aging. Neuroscience.

[9] Mostany R (2013). Altered synaptic dynamics during normal brain aging. J. Neurosci..

[10] Edison P (2007). Amyloid, hypometabolism, and cognition in Alzheimer disease: An [11C]PIB and [18F]FDG PET study. Neurology.

[11] Kalpouzos G (2009). Voxel-based mapping of brain gray matter volume and glucose metabolism profiles in normal aging. Neurobiol. Aging.

[12] Franke TN (2020). In vivo imaging with (18)F-FDG- and (18)F-Florbetaben-PET/MRI detects pathological changes in the brain of the commonly used 5XFAD mouse model of Alzheimer's disease. Front. Med. (Lausanne).

[13] Zou QH (2008). An improved approach to detection of amplitude of low-frequency fluctuation (ALFF) for resting-state fMRI: Fractional ALFF. J. Neurosci. Methods.

[14] Aiello M (2015). Relationship between simultaneously acquired resting-state regional cerebral glucose metabolism and functional MRI: A PET/MR hybrid scanner study. Neuroimage.

[15] Biswal B, Yetkin FZ, Haughton VM, Hyde JS (1995). Functional connectivity in the motor cortex of resting human brain using echo-planar MRI. Magn. Reson. Med..

[16] Pifferi F (2018). Caloric restriction increases lifespan but affects brain integrity in grey mouse lemur primates. Commun. Biol..

[17] Sawiak SJ, Picq JL, Dhenain M (2014). Voxel-based morphometry analyses of in vivo MRI in the aging mouse lemur primate. Front. Aging Neurosci..

[18] Kraska A (2011). Age-associated cerebral atrophy in mouse lemur primates. Neurobiol. Aging.

[19] Djelti F (2016). Impaired fasting blood glucose is associated to cognitive impairment and cerebral atrophy in non-human primates. Aging.

[20] Aujard F (2001). Effect of ambient temperature on the body temperature rhythm of male gray mouse lemurs (*Microcebus murinus*). Int. J. Primatol..

[21] Celestine M, Nadkarni NA, Garin CM, Bougacha S, Dhenain M (2020). Sammba-MRI: A library for processing SmAll-MaMmal Br Ain MRI data in python. Front. Neuroinform..

[22] Gorgolewski K (2011). Nipype: A flexible, lightweight and extensible neuroimaging data processing framework in python. Front. Neuroinform..

[23] Cox RW (1996). AFNI: Software for analysis and visualization of functional magnetic resonance neuroimages. Comput. Biomed. Res. Int. J..

[24] Oguz I, Zhang H, Rumple A, Sonka M (2014). RATS: Rapid automatic tissue segmentation in rodent brain MRI. J. Neurosci. Methods.

[25] Garin CM (2021). Resting state functional atlas and cerebral networks in mouse lemur primates at 11.7 Tesla. Neuroimage.

[26] Carp J (2012). The secret lives of experiments: Methods reporting in the fMRI literature. Neuroimage.

[27] Esteban O (2019). fMRIPrep: A robust preprocessing pipeline for functional MRI. Nat. Methods.

[28] Mandino F (2019). Animal functional magnetic resonance imaging: Trends and path toward standardization. Front. Neuroinform..

[29] Zuo XN (2010). The oscillating brain: Complex and reliable. Neuroimage.

[30] Jia XZ (2020). Percent amplitude of fluctuation: A simple measure for resting-state fMRI signal at single voxel level. PLoS ONE.

[31] Nadkarni NA, Bougacha S, Garin C, Dhenain M, Picq JL (2019). A 3D population-based brain atlas of the mouse lemur primate with examples of applications in aging studies and comparative anatomy. Neuroimage.

[32] Abraham A (2014). Machine learning for neuroimaging with scikit-learn. Front. Neuroinform..

[33] Woo CW, Krishnan A, Wager TD (2014). Cluster-extent based thresholding in fMRI analyses: Pitfalls and recommendations. Neuroimage.

[34] Lacquaniti F, Guigon E, Bianchi L, Ferraina S, Caminiti R (1995). Representing spatial information for limb movement: Role of area 5 in the monkey. Cereb. Cortex.

[35] Hu S, Chao HH, Zhang S, Ide JS, Li CS (2014). Changes in cerebral morphometry and amplitude of low-frequency fluctuations of BOLD signals during healthy aging: Correlation with inhibitory control. Brain Struct. Funct..

[36] Gardner EP, Babu KS, Ghosh S, Sherwood A, Chen J (2007). Neurophysiology of prehension. III. Representation of object features in posterior parietal cortex of the macaque monkey. J. Neurophysiol..

[37] Caminiti R (2010). Understanding the parietal lobe syndrome from a neurophysiological and evolutionary perspective. Eur. J. Neurosci..

[38] Le Brazidec M (2017). How aging affects grasping behavior and pull strength in captive gray mouse lemurs (*Microcebus murinus*). Int. J. Primatol..

[39] Zhao J (2019). Low-frequency fluctuation characteristics in rhesus macaques with SIV infection: A resting-state fMRI study. J. Neurovirol..

[40] Rao JS (2014). Fractional amplitude of low-frequency fluctuation changes in monkeys with spinal cord injury: A resting-state fMRI study. Magn. Reson. Imaging.

[41] Wu TL (2016). Effects of isoflurane anesthesia on resting-state fMRI signals and functional connectivity within primary somatosensory cortex of monkeys. Brain Behav..

[42] Chang WT (2018). Connectomic imaging reveals Huntington-related pathological and pharmaceutical effects in a mouse model. NMR Biomed..

[43] Yan CG (2017). Aberrant development of intrinsic brain activity in a rat model of caregiver maltreatment of offspring. Transl. psychiatry.

[44] Li J (2018). Effects of stress on behavior and resting-state fMRI in rats and evaluation of Telmisartan therapy in a stress-induced depression model. BMC Psychiatry.

[45] Garin CM (2022). An evolutionary gap in primate default mode network organization. Cell Rep..

